# Dendritic sodium spikes endow neurons with inverse firing rate response to correlated synaptic activity

**DOI:** 10.1007/s10827-018-0707-7

**Published:** 2018-12-13

**Authors:** Tomasz Górski, Romain Veltz, Mathieu Galtier, Hélissande Fragnaud, Jennifer S. Goldman, Bartosz Teleńczuk, Alain Destexhe

**Affiliations:** 1 Unité de Neurosciences, Information et Complexité, Centre National de la Recherche Scientifique, Gif-sur-Yvette, France; 2 European Institute for Theoretical Neuroscience, Paris, France; 3 Inria, Sophia Antipolis, France

**Keywords:** Dendritic integration, Synaptic input correlations

## Abstract

Many neurons possess dendrites enriched with sodium channels and are capable of generating action potentials. However, the role of dendritic sodium spikes remain unclear. Here, we study computational models of neurons to investigate the functional effects of dendritic spikes. In agreement with previous studies, we found that point neurons or neurons with passive dendrites increase their somatic firing rate in response to the correlation of synaptic bombardment for a wide range of input conditions, i.e. input firing rates, synaptic conductances, or refractory periods. However, neurons with active dendrites show the opposite behavior: for a wide range of conditions the firing rate decreases as a function of correlation. We found this property in three types of models of dendritic excitability: a Hodgkin-Huxley model of dendritic spikes, a model with integrate and fire dendrites, and a discrete-state dendritic model. We conclude that fast dendritic spikes confer much broader computational properties to neurons, sometimes opposite to that of point neurons.

## Introduction

Increasing evidence shows that nonlinear integration of synaptic inputs in dendrites is crucial for the computational properties of neurons. A major role in the integration is played by dendritic spikes: regenerative currents through Na^+^, Ca^2+^ or NMDAr channels. The first evidence of dendritic spikes came from field recordings (Cragg and Hamlyn 1955; Andersen 1960; Fujita and Sakata 1962; Eccles et al. 1966; Llinás et al. 1968), corroborated by intracellular recordings (Llinas and Nicholson 1971; Houchin 1973; Wong et al. 1979). The repertoire of techniques was further enlarged by patch clamp (Stuart et al. 1994; Magee and Johnston 1995a, 1995; Spruston et al. 1995a, 1995) and optical methods. Calcium imaging allowed for the direct observation of calcium spikes (Jaffe et al. 1992; Miyakawa et al. 1992; Regehr et al. 1989; Regehr and Tank 1990; 1992), while glutamate uncaging and voltage sensitive dyes led to the discovery of NMDA spikes (Schiller et al. 2000; Polsky et al. 2004).

Dendritic spikes allow for more subtle integration of synaptic input than in a passive dendrite. A single dendritic branch can act as a coincidence detector, generating a spike when exposed to synchronized input (Williams and Stuart 2002; Losonczy and Magee 2006; Polsky et al. 2004). The propagation of dendritic spikes generated in the distal part of a dendritic tree can be gated by synaptic input in the proximal region, as was shown for hippocampal CA1 pyramidal neurons (Jarsky et al. 2005), and L5 pyramidal neurons (Larkum et al. 2001). After the initiation of a dendritic spike, sodium channels inactivate and the branch switches into a refractory state which crucially affects integration (Remy et al. 2009). Backpropagating action potentials also play an essential role in spike time-dependent plasticity (Magee et al. 1997; Bi and Poo 1998; Markram et al. 1997), and the participation of local dendritic spikes has been implicated in long-term potentiation (Golding et al. 2002; Kim et al. 2015).

Sodium spikes can propagate in many cell types: neocortical pyramidal cells (Kim and Connors 1993; Stuart et al.^1994^; Larkum et al. 1999a, 1999; Zhu 2000) hippocampal CA1 and CA3 pyramidal cells (Turner et al. 1991; Wong and Stewart 1992; Andreasen and Lambert 1995; Golding and Spruston 1998; Golding et al. 1999) and interneurons (Martina et al. 2000), or thalamic neurons (Williams and Stuart 2000). Models predicted that, in vivo, the presence of synaptic background activity should greatly enhance the initiation and propagation of dendritic spikes (Rudolph et al. 2001; Destexhe et al. 2003). This suggests that there can be a heavy traffic of dendritic spikes, as indeed found in recent dendritic recordings in awake animals (Moore et al. 2017). Therefore, interactions between dendritic spikes likely play an important role in dendritic integration.

These interactions between spikes can be conveyed by the refractory period which follows each spike (Hodgkin and Huxley 1952), and can prevent initiations of subsequent spikes, or can result in a collision and annihilation of spikes (Bullock 1945). Here, comparing different types of computational models, we show that interactions between dendritic spikes can change the way the correlation of synaptic input affects the firing rate of the cell. For single-compartment neurons and neurons with passive dendrites, an increase of correlation of synaptic input is known to cause an increase of firing rate, while this relation can be reversed only for high input intensities (Bernander et al. 1994; Murthy and Fetz 1994; De La Rocha et al. 2007; Shea-Brown et al. 2008). We show that neurons with active dendrites behave the opposite way: for a wide range of input conditions, the firing rate varies inversely proportionally to the level of correlation. We discuss possible consequences of this property at the network level.

## Results

We first show the phenomenon of inverse correlation processing using a multi-compartment Hodgkin-Huxley model with active dendrites. Next, we investigate the impact of the refractory period duration on inverse processing using a multi-compartment integrate and fire model. Finally, we show that this phenomenon is also present in simplified discrete-state dendritic models.

### Model of correlated synaptic activity

In all models studied here, we considered neurons subjected to in vivo–like synaptic activity. In particular, we aimed at investigating the effects of synaptic correlations on the firing of the cell. The synapses were located on the somatic and dendritic compartments and were of two types: excitatory AMPA synapses (with reversal potential *E*_*e*_ = 0mV) and inhibitory GABA_*A*_ synapses (*E*_*i*_ = − 75mV), with five times more excitatory than inhibitory synapses (DeFelipe and Fariñas 1992).

We considered the case where the pre-synaptic spikes triggering the excitatory inputs are correlated. There might be two biological sources of such correlation. First of all, in cortical networks, neurons are known to share some of their synaptic inputs which inevitably makes their spikes correlated. Since this type of correlation is common to larger populations of neurons, we call it the global correlation (Renart et al. 2010). In addition, a single axon can create several synapses on close-by segments of the dendrite activating them at the same time (Sorra and Harris 1993), so that the correlation of the input spikes within the same dendritic segments (local correlation) can be higher than the global correlation.

**Fig. 1:**
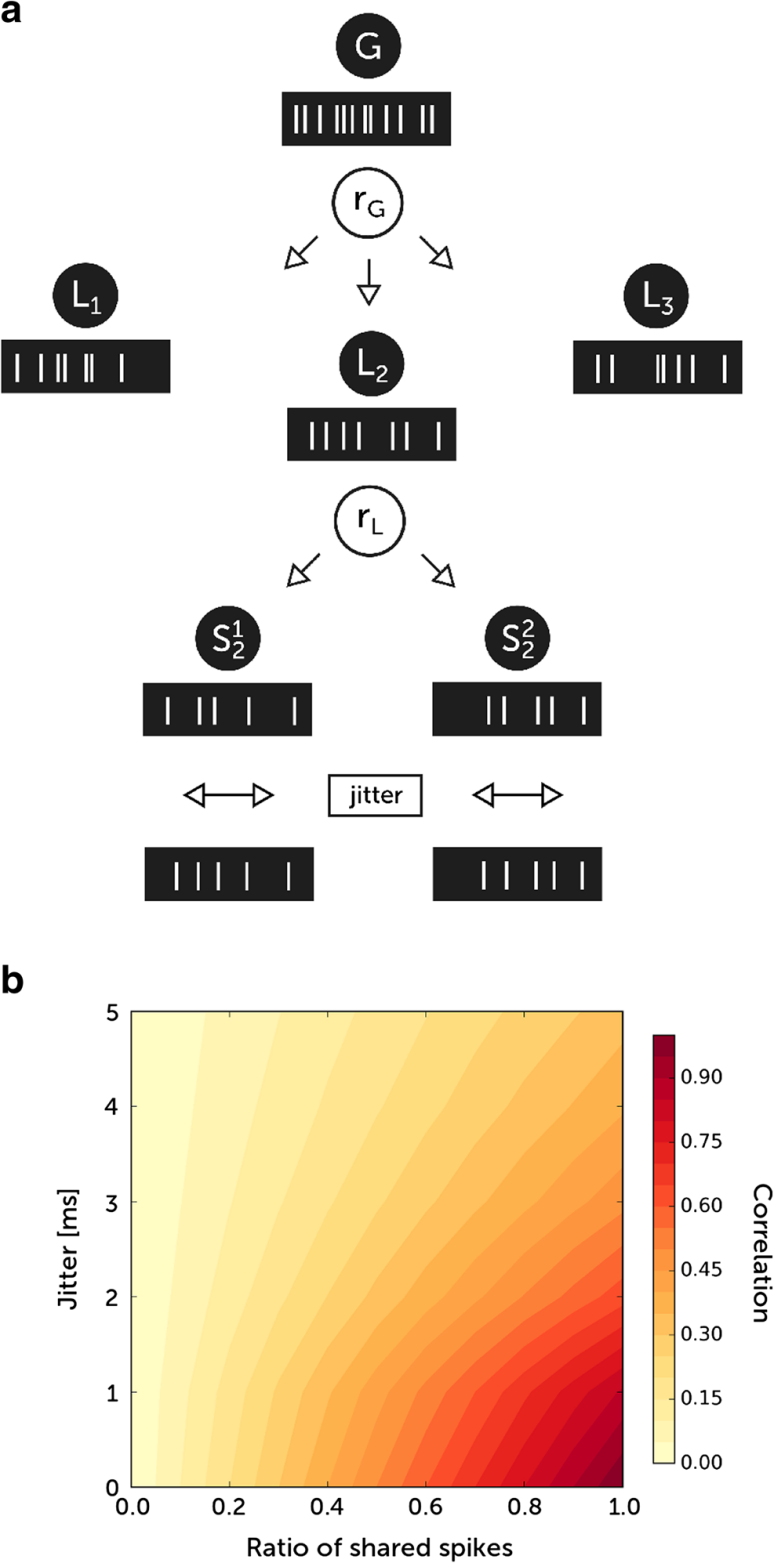
Model of correlated synaptic activity. **a** From the global spike train *G* spike times are distributed with probability *r*_*G*_ to the local spike trains *L*_*k*_ corresponding to compartments. From the local spike trains, spikes are distributed with probability *r*_*L*_ to synapses ${S_{k}^{i}}$. The spike times are then shifted in time with random jitter. **b** The dependence of correlation measure ${C}_{{\Delta } t}^{ij}$ between two spike trains *i* and *j* on a ratio of shared spikes and on the jitter. To calculate the correlation, the time range of the cross-covariance function integral was Δ*t* = 2 ms. The mean rate of the process was 20 Hz, and its duration was 50 s

To generate such locally and globally correlated synaptic inputs, we used a previously proposed algorithm (Brette 2009). From a global spike train *G* obtained from Poisson process, we draw random spikes with probability *r*_*G*_. The spike train *L*_*k*_ thus obtained corresponds to all synapses on a single dendritic compartment. Finally, the spike train ${S_{k}^{i}}$ for a synapse *i* situated on compartment *k* is built by drawing spikes from *L*_*k*_ with local probability *r*_*L*_ [Fig. 1].

In this model, the ratio of shared spikes between synapses situated on the same compartment is equal to *c*_*L*_ = *r*_*L*_ and the ratio of spikes shared by synapses situated on different compartments is equal to *c*_*G*_ = *r*_*L*_*r*_*G*_. Note that *c*_*L*_ is always greater than or equal to *c*_*G*_. At the end, we added a jitter to each spike time to obtain desynchronized input. The jitter is drawn from exponential distribution with a standard deviation *τ*_*j*_ with equal probability for positive and negative values. To obtain the time-dependent synaptic conductances, we convolve the resulting spike trains with an exponential function reflecting the change of synaptic conductance due to a single spike.

### Response to correlated synaptic activity in biophysical dendritic models

**Fig. 2:**
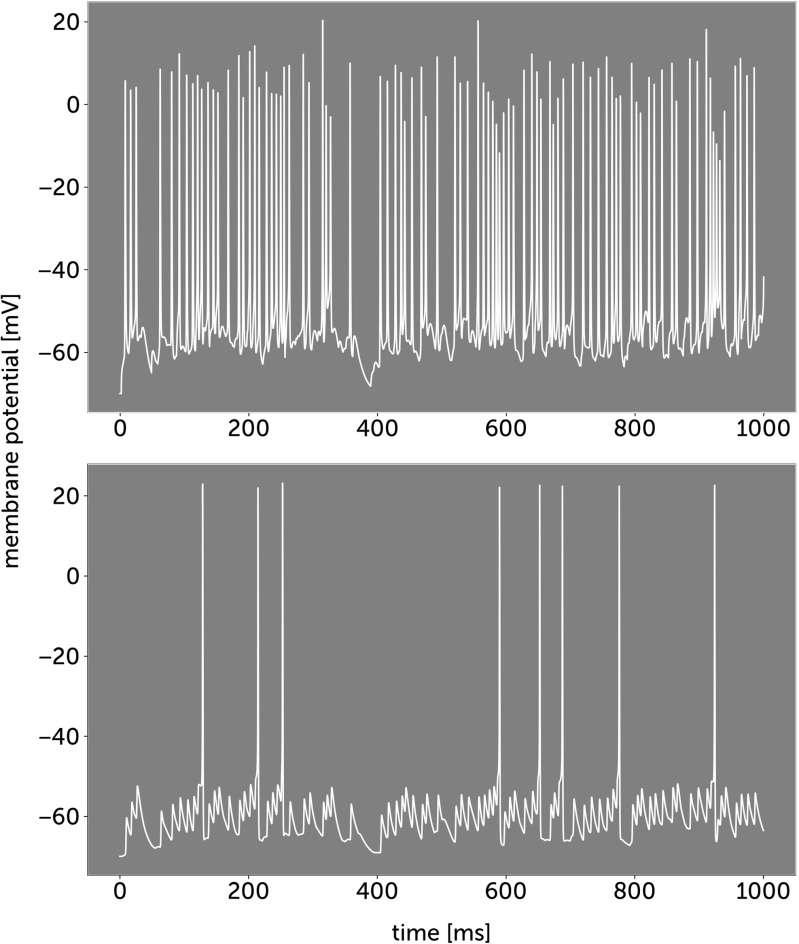
Comparison of dendritic and somatic spikes. **a** Membrane voltage in the dendrite 500 *μ* m from the soma. **b** Membrane voltage in the soma. Most dendritic spikes cannot actively invade the soma though they cause depolarizations with amplitudes of just a few millivolts

**Fig. 3:**
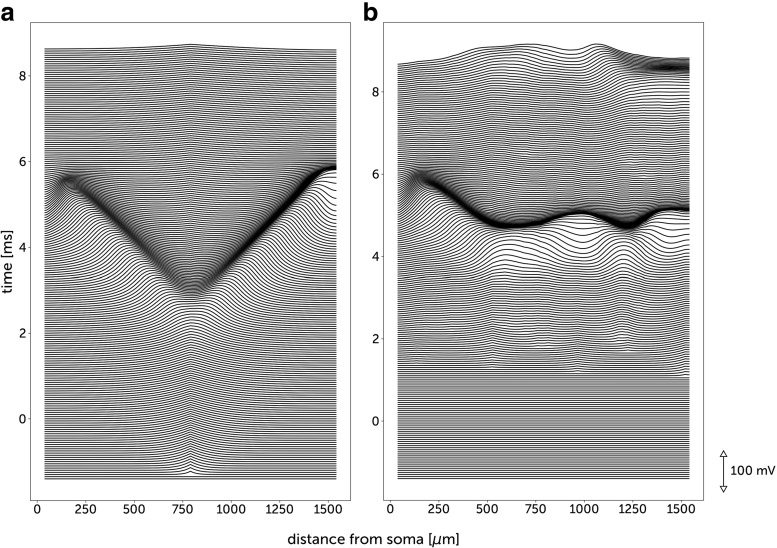
Propagation and collision of dendritic spikes in a Hodgkin-Huxley model. Each line represents the profile of membrane voltage at a given instant. **a** A sodium spike is created near the center of the dendrite and propagates toward the soma and the distal dendritic end. **b** The correlated synaptic bombardment (*c*_*G*_ = 0.1, *τ*_*j*_ = 5ms) creates two sodium spikes (around 600 *μ* m and 1200 *μ* m from soma) which wavefronts collide and cancel due to the refractory period

We first study correlation processing in a Hodgkin-Huxley model of neurons with dendrites (see Methods). In our model the dendritic spikes can be one order of magnitude more frequent than somatic spikes (Fig. 2), this ratio is similar to that observed in vivo (Moore et al. 2017). In Fig. 3, we show how dendritic sodium spikes propagate and collide in the model. We investigate how interactions between dendritic spikes can affect the response of a neuron to correlated synaptic activity.

**Fig. 4:**
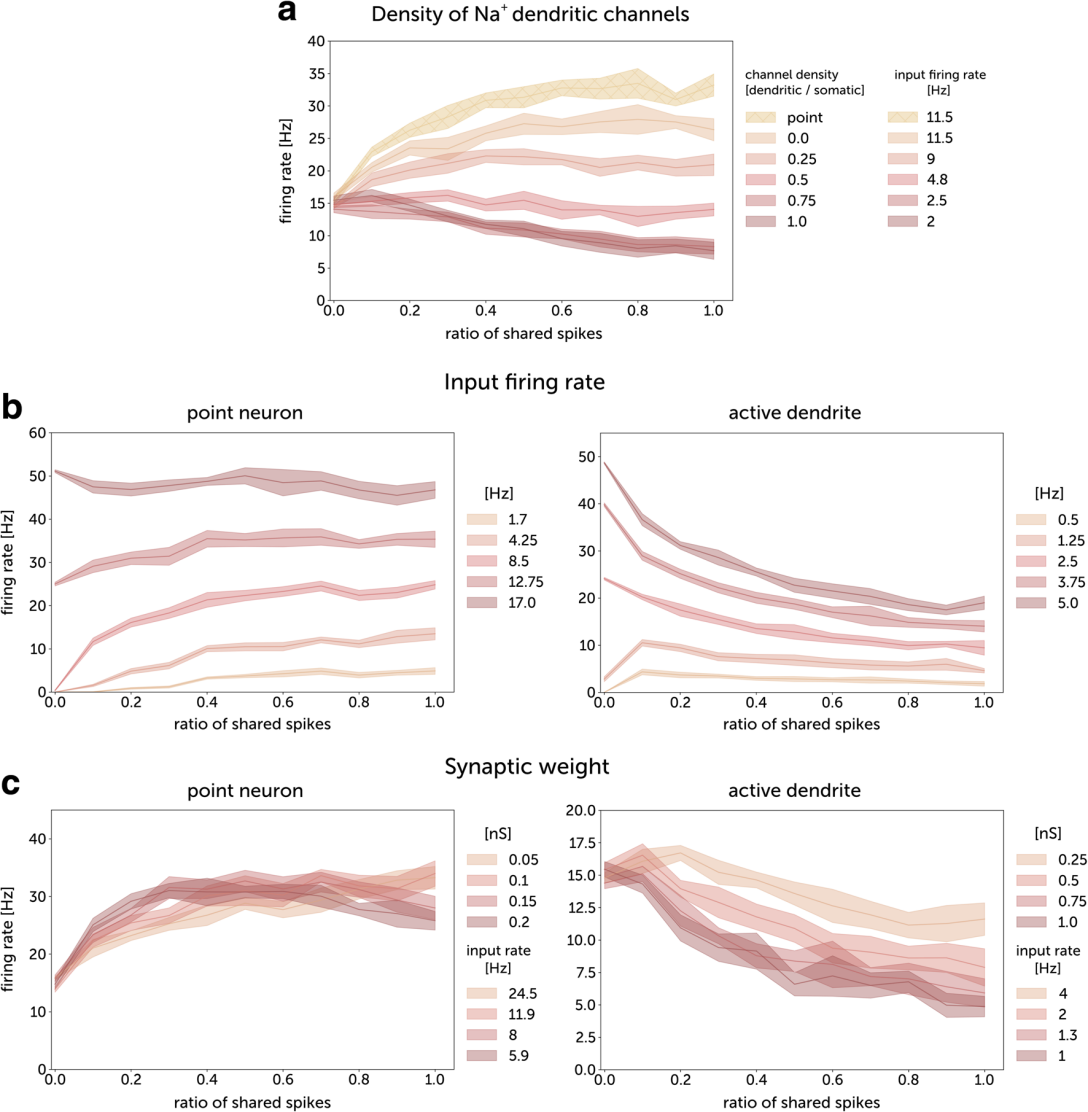
Somatic firing rate responses to correlated synaptic activity for a Hodgkin-Huxley point neuron and for a Hodgkin-Huxley neuron with dendrite. **a** Firing responses for different densities of sodium channels in a dendrite. While changing dendritic densities of sodium channels, the somatic densities were left unchanged (12 mS/cm^2^ for Na^+^ conductance and 7 mS/cm^2^ for K^+^ conductance. The input firing rates were adjusted to dendritic channel densities to obtain the same somatic firing rate for uncorrealed input. Responses of neurons with dendrites are compared with the responses of point neurons with scaled synapses. The synaptic conductances were 0.5 nS for the neuron with dendrite, and 0.105 nS for the point neuron. **b** Firing responses for different input firing rates for a Hodgkin-Huxley point neuron with scaled synapses and for a Hodgkin-Huxley neuron with dendrite. **c** Firing responses for different synaptic conductances for point neurons and neurons with dendrites. *For plots (b) and (c):* the dendritic Na^+^ channel density of the multi-compartment neuron was equal to the somatic channel density (12 mS/cm^2^). *For all plots:* in each of the 200 dendritic compartments, there was one excitatory synapse; 40 inhibitory synapses were located on the soma. For the point neuron, all 200 excitatory synapses were placed on the soma. The ratio of shared spikes for the multicompartment neuron is therefore c_*G*_. The time constant of jitter for the generation of correlated input spike trains, *τ*_*j*_ was 10 ms

We run simulations of the model under correlated synaptic activity. For each run, we measured the firing rate of somatic spikes and asked how this quantity is affected by the ratio of shared spikes (Fig.4). For each ratio we performed 20 runs with a duration of 20 s. With increasing density of active channels in the dendrite, the somatic firing response changes to become *inverse*, i.e. we observe a decrease of somatic firing rate with increasing correlation of synaptic input (Fig.4a). To adequately compare the multicompartment neuron with a point neuron, we have scaled the synaptic conductances in the point neuron to emulate filtering of EPSPs by the dendrite. The inverse response of a neuron with a dendrite can be enhanced by the increase of input firing rate and the increase of synaptic weights (Fig.4a, b). In contrast, in the point neuron model, the firing rate response generally increased with correlation, as found previously (Bernander et al. 1994; Murthy and Fetz 1994). The firing rate could also remain approximately constant for high values of the synaptic input (> 40 Hz). The increase of synaptic weights resulted in a more pronounced inverse response to correlation of synaptic input. The reason for this effect is that, for low weights, more synaptic potentials remain sub-threshold and summed synaptic inputs from single or multiple compartments are necessary to trigger a single spike. Hence, a higher input correlation leads to an increase in the probability of generating a spike. On the other hand, for larger synaptic weights, few synaptic inputs may trigger a spike, whose propagation to the soma is limited by the effects of the refractory period and collisions. In this regime, a higher correlation means higher probability of stopping spike propagation by the refractory periods of preceding spikes. Therefore, we observe a stronger decrease in the somatic firing rate.

**Fig. 5:**
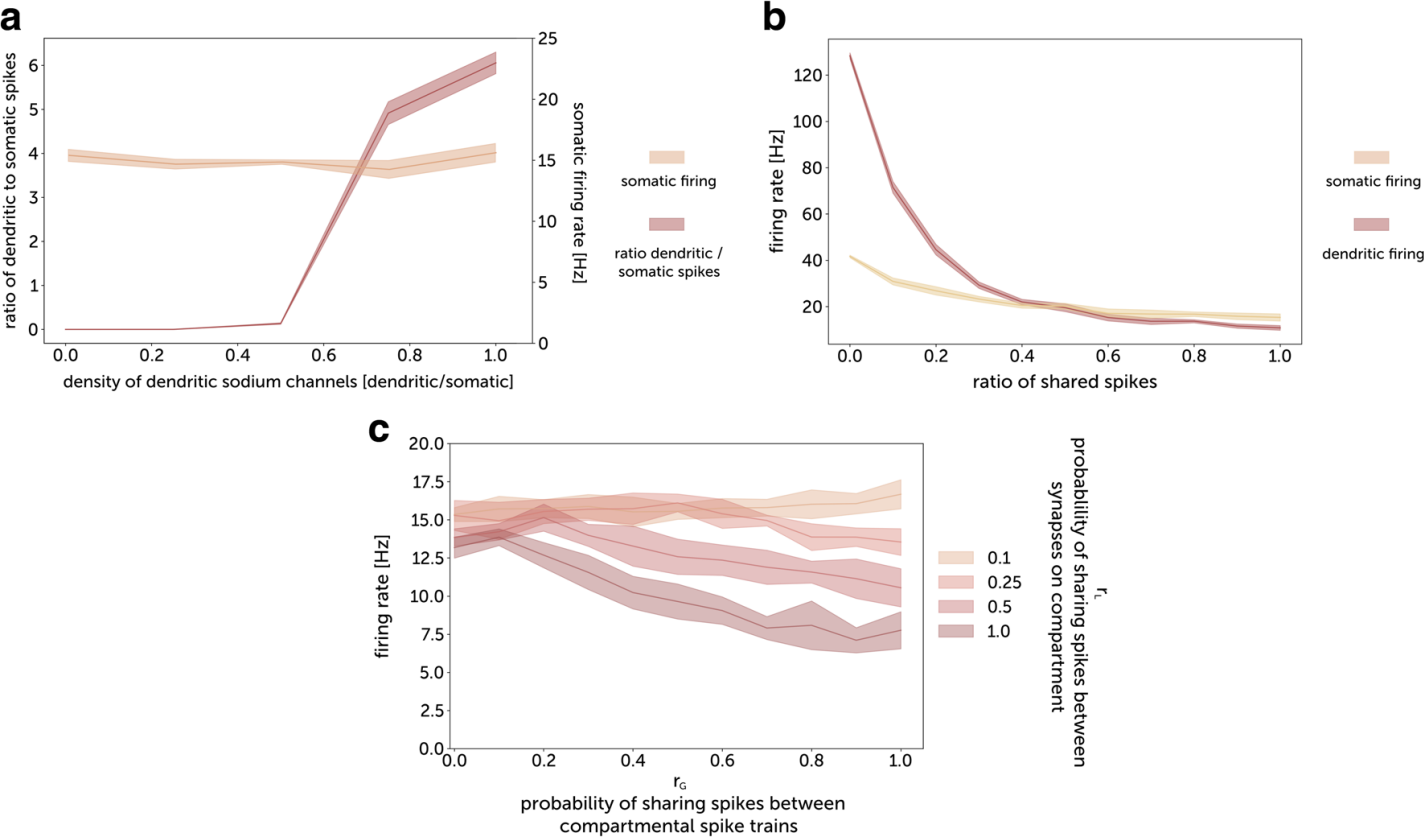
Somatic and dendritic spikes. **a** Ratio of dendritic spike firing to somatic spike firing as a function of the density of dendritic sodium channels. The somatic firing rate was kept at a constant level of 15 Hz by adjusting the input firing rate while changing channel densities. The input spike trains were uncorrelated. The dendritic spikes were detected in the middle of the dendrite 500 *μm* from the soma. **b** Somatic and dendritic firing responses to correlated synaptic activity. The density of dendritic sodium channels was 12 mS/cm^2^ and the input firing rate per synapse was 4 Hz. **c** Somatic firing response to global correlation r_*G*_ for different values of local correlation r_*L*_. The dendrite consisted of 50 compartments of size 20 *μm*, with 4 excitatory synapses on each compartment yielding the same total number of synapses as for the previous simulations. *For all plots*: The synaptic conductances were 0.5 nS. The time constant of jitter for the generation of correlated input spike trains, *τ*_*j*_ was 10 ms

To investigate the mechanism of inverse processing, we measured the frequency of dendritic spikes simultaneously with the frequency of somatic spikes. With an increase of the density of dendritic sodium channels, the ratio of dendritic to somatic spikes increases abruptly, reaching values around 10 for the same density of somatic and dendritic channels (Fig. 5a). With an increase of synaptic input correlation, the number of dendritic spikes decreases due to spike cancellations, which results in a weaker depolarization of the soma and a lower somatic firing rate (Fig. 5b.)

We checked how local correlations affect the firing rate response (Fig. 5c). We observed that neurons with active dendrites display a stronger inverse response to global correlations for more locally synchronized synaptic activity. Hence increasing local correlations acts as increasing effective synaptic weights (cf. Fig. 4c). This interplay between local and global correlation can largely affect the properties of dendritic integration.

To investigate the impact of the refractory period duration on inverse processing, we considered an integrate and fire type model. In particular, we used a multi-compartment exponential integrate and fire model, which can be extended to include the neuronal adaptation (Brette and Gerstner 2005). We extended the exponential integrate and fire model with axial currents and spike waveform to adapt it for modeling multi-compartment dendrites (see Methods for the equations of the multi-compartment model). With this modification, the model can produce spikes propagating across the tapered linear dendrite, but the propagation can be stopped by the tunable refractory period caused by other spikes.

The morphology of the integrate and fire model was the same as for the Hodgkin-Huxley model (see Methods). In the same way as for the Hodgkin-Huxley model, we measure the firing rate of somatic spikes as a function of the ratio of shared spikes.

**Fig. 6:**
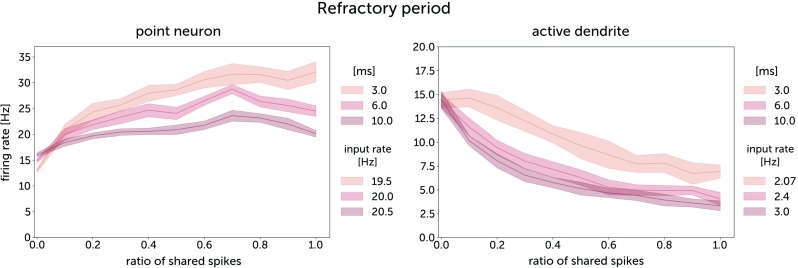
Somatic firing rate responses to correlated synaptic activity for an integrate-and-fire point neuron and for an integrate-and-fire neuron with a dendrite. The synaptic conductances were 0.5 nS for a neuron with a dendrite, and 0.105 nS for a point neuron. The time constant of jitter for the generation of correlated input spike trains, *τ*_*j*_ was 10 ms

For neurons with dendrites, increasing the duration of the refractory period results in a stronger inverse response to correlation (Fig. 6), while for the point neurons, the response remained positive even for 10 ms refractory period.

### Simplified discrete-state dendritic model

**Fig. 7:**
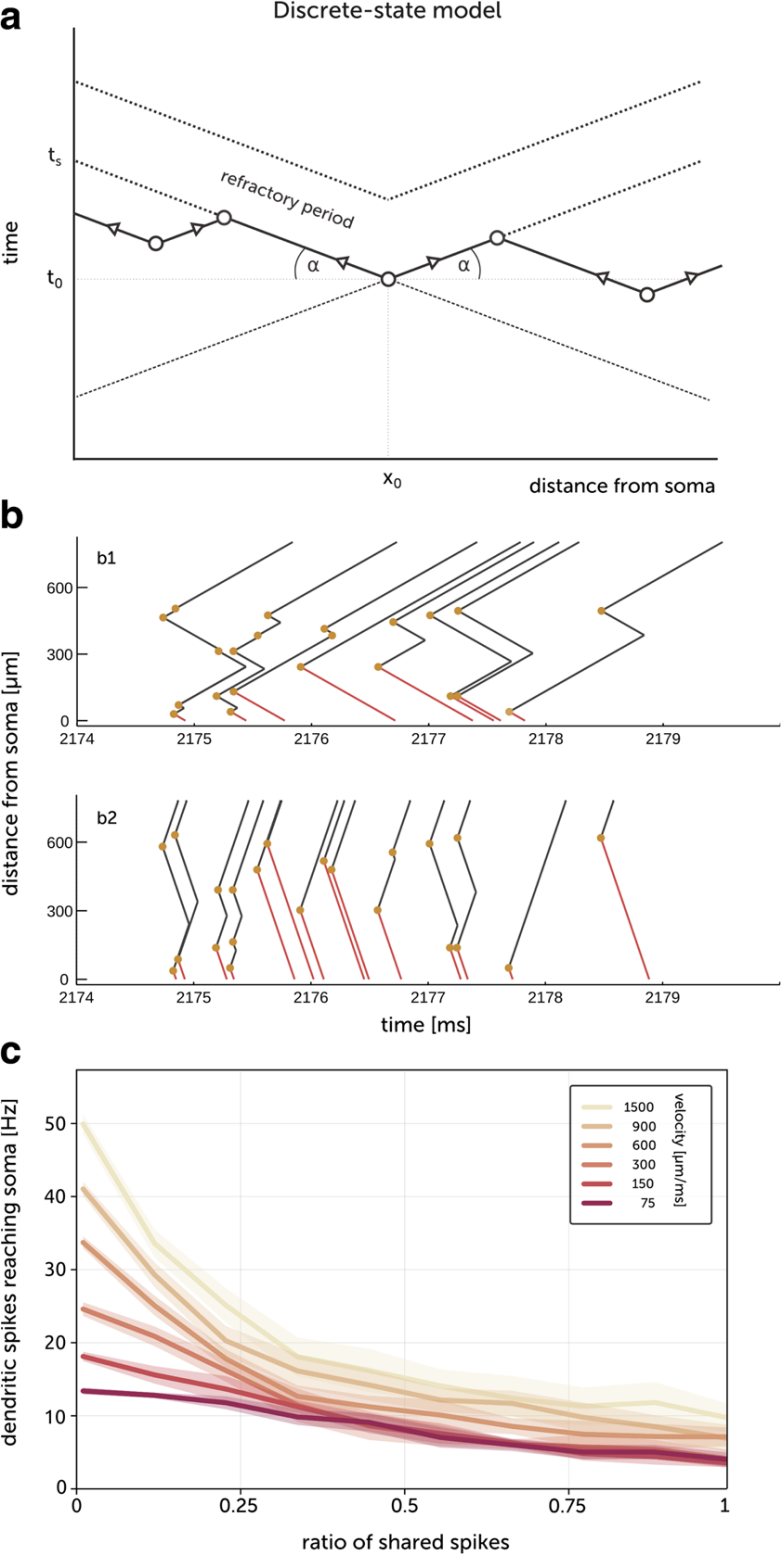
Discrete state dendritic model. **a** Illustration of propagations and collisions of dendritic spikes. The dendritic spike is created at point (*t*_0_, *x*_0_) and propagates to the soma and to the distal end of a dendrite. Spikes created in the area enclosed between the wavefronts and their extensions can collide with the spike created at point (*t*_0_, *x*_0_). Moreover, spikes cannot be created in the refractory area. The velocity of the spike is $v_{s} = \text {cot} \alpha = \frac {x_{0}}{t_{s} - t_{0}} = \frac {L - x_{0}}{t_{d} - t_{0}}$, where *L* is a length of a dendrite and *t*_*s*_ and *t*_*d*_ are the times when the spike reaches soma and distal end of a dendrite respectively. The spikes created in space-time area $P_{col} = \frac {{x_{0}^{2}}}{v_{s}} + \frac {(L - x_{0})^{2}}{v_{s}}$ can collide with the spike created at point (*t*_0_, *x*_0_). The space-time area in a refractory state is *P*_*ref*_ = *Lt*_*r*_. Assuming that a density of probability of spike creation *p*_*s*_(*t*, *x*) is uniform and assuming normalization ${{\int }_{0}^{T}} dt {{\int }_{0}^{L}}dx \ p_{s}(t,x) = N$, where *N* is a total number of spikes created in time *T*, the number of collisions should be proportional to *p*_*s*_*P*_*col*_ and increases linearly with the length of the dendrite *L*, while the number of spikes not created due to refractory period *p*_*s*_*P*_*ref*_ doesn’t scale with *L*. **b** Propagations of spikes in a discrete-state dendritic model. The origins of spikes are marked by yellow circles. Red lines indicate dendritic spikes which reach the soma. The velocity of dendritic spikes is 4 times higher in (b2) than in (b1). The ratio of shared input spikes, *c*_*G*_ = 0.5. **c** Dependence of the mean rate of dendritic spikes reaching the soma on global correlation for the discrete-state model for different velocities of the dendritic spikes. Time constant of the jitter *τ*_*j*_ = 2 ms

The simple space-time illustration of collisions of dendritic spikes is given in Fig. 7a. We can distinguish two modes of interference between spikes: 1) direct collisions 2) inability to initiate or propagate spike due to refractory period. Assuming a constant velocity of propagation of the dendritic spikes, a uniform distribution of dendritic spikes and the same total number of spike creations in a dendrite, the number of direct collisions is proportional to the length of the dendrite, while the number of spikes prevented by the refractory period does not increase with the length of a dendrite, see Fig. 7a.

Finally, we designed a mathematical model, called *discrete-state model*, aiming at grasping the core mechanism of spike propagation and annihilation. Compared to the more detailed models described so far, the discrete-state model has much smaller simulation time and allows to easily modify parameters independently (e.g. propagation speed). The simulation is exact in that it is not based on a (time) discretization of the propagation of dendritic fronts. Finally, it allows to see whether the annihilation of spikes is enough to explain the decrease of mean somatic spikes. More precisely, given a set of spatio-temporal synaptic inputs $S=\{(t_{i},x_{i})\}_{i = 1}^{N}$, assume that each input (*t*_*i*_, *x*_*i*_) produces two contra-propagating fronts (e.g. dendritic spikes). *S* is generated with the procedure described in Section 2.1.

In Fig. 7b, the synaptic inputs are the yellow dots and the lines, the propagating fronts. Starting from *S*, we build the set of fronts and annihilation events recursively (see Methods). Figure 7b shows the networks of propagating fronts for different propagation speeds but the same inputs *S*. One can see that increasing speed increases the dendritic spikes reaching the soma (*x* = 0). Intuitively, this occurs because a front has less chance to meet another front for higher propagation speeds. Finally, in Fig. 7c we present the dependency of the mean number of spike reaching the soma as a function of the ratio of shared spikes. In this scenario, each synaptic input triggers a dendritic spikes, so the result corresponds to the case of large synaptic weights of the multi-compartment Hodgkin-Huxley model (Fig. 4c).

## Discussion

In this paper, we have investigated the behavior of excitable neuronal dendrites using different models. The main property that we explored is the ability of the dendrite to initiate and propagate fast dendritic spikes and resulting consequences for processing correlated synaptic activity.

One of our main findings is that the presence of fast dendritic spikes endow neurons with the ability to process inputs according to a mode of inverse correlation processing, in which the neuron fires less when the inputs are more correlated. This is contrary to point neurons, which generally show the opposite mode, where firing is directly increased by correlations (Bernander et al. 1994; Murthy and Fetz 1994; De La Rocha et al. 2007; Shea-Brown et al. 2008). However, point neurons can display inverse correlation processing only for very long refractoriness or for extreme stimulation conditions. In contrast, active dendrite display inverse correlation processing for a much broader extent of parameter space.

We have further shown that this inverse correlation processing mode is present in different models, such as a Hodgkin-Huxley model of fast sodium dendritic spikes, an integrate and fire model for dendrites, or a discrete-state dendritic model. We discuss below possible implications of these findings, and ways to test them experimentally.

Our model challenges the well-known finding that the presence of correlations in the pre-synaptic activity necessarily augments the firing rate of neurons (Bernander et al. 1994; Murthy and Fetz 1994; De La Rocha et al. 2007; Shea-Brown et al. 2008). We find here that neurons equipped with excitable dendrites can operate in a regime which is opposite: neurons with active dendrites generally decrease their firing when correlations are present in their afferent activity. Furthermore, this inverse correlation processing mode is dominant in models where dendrites generate fast spikes (such as Na^+^ spikes) whenever the intensity of synaptic bombardment is high enough. Recent experiments (Moore et al. 2017) provided strong evidence that Na^+^ dendritic spikes are much more frequent than somatic spikes in awake animals (10 times more frequent during exploration), so there is a serious possibility that neurons can be set to this inverse correlation processing mode.

It is interesting to ask how the response to synaptic input correlation can be affected by the morphology of a dendritic tree. In our linear model of a dendrite, the length of the distal dendritic part is much shorter than the total length of distal branches present in more complex morphologies. The initiation of a sodium spike is enhanced in more distal branches where higher input resistance causes higher local depolarization triggered by a single synaptic input. The resulting increase of the spike rate in distal dendrites can lead to the higher rate of spike annihilations and therefore amplified inverse correlation processing. Indeed, the collisions of dendritic spikes can effectively prevent the propagation of sodium spikes from distal branches to the soma (Royer and Miller 2007).

While it is beyond the scope of the present study, one can ask if NMDA and Ca^2+^ spikes may have similar impact on correlation processing. The NMDA spike, however, is different than the Na^+^ spike because it cannot usually propagate (it would require continuous glutamatergic stimulation along the dendrite). Therefore interactions between NMDA spikes should not be as global as interactions between Na^+^ spikes. Calcium spikes on the other hand usually propagate in a restricted area of the apical dendritic tree and are not likely to actively invade the soma (Schiller et al. 1997), moreover their frequencies are much lower than the frequencies of fast sodium spikes (Golding et al. 1999; Moore et al. 2017).

We demonstrated the inverse correlation phenomenon also on a simplified integrate and fire type model. Although this type of model is usually applied to point neurons without any spatial extent, we extended it over multiple compartments connected through axial currents such that propagating spikes can be simulated. The model used here can be extended to include neuronal adaptation (yielding the AdEx model). The AdEx model allows to simulate different intrinsic properties such as bursting and adaptation, (Brette and Gerstner 2005; Destexhe 2009). This allows us to later extend the present model by including such mechanisms in dendrites. The AdEx model is also compatible with neuromorphic hardware (Schemmel et al. 2010), which is presently extended to include neurons with excitable dendrites (Schemmel et al. 2017). In addition, to allow for analytical treatment of the dendritic spikes, we further simplified the model to a discrete state model capturing the dynamics of the collisions of dendritic spikes.

What are the consequences of such inverse correlation processing? One obvious effect will be to cancel correlations at the network level, because the neurons subject to correlated input will fire less, and neurons with decorrelated input will dominate the population, and will most likely shift ensembles towards decorrelated firing. Such dynamics should be examined by future network models using neurons equipped with excitable dendrites.

Finally, it will be important to directly test inverse correlation processing experimentally. Dynamic-clamp was used previously to investigate the fact that neurons increase their synchrony (Reyes 2003), but unfortunately, this technique cannot be used to test the present mechanism, because dynamic-clamp emulates inputs in the soma (the site of the recording electrode), while it is important here that the inputs are dendritic. One possibility would be to use 2-photon imaging with glutamate uncaging (Branco et al. 2010) to simulate inputs with controlled frequency and synchrony. Another possibility would be to use voltage-sensitive dye imaging of dendrites in vitro (Zecevic 1996) combined with controlled network activity in the slice, to directly monitor the genesis, propagation and possible collision of dendritic spikes.

## Methods

### Hodgkin-Huxley dendritic model

**Fig. 8:**
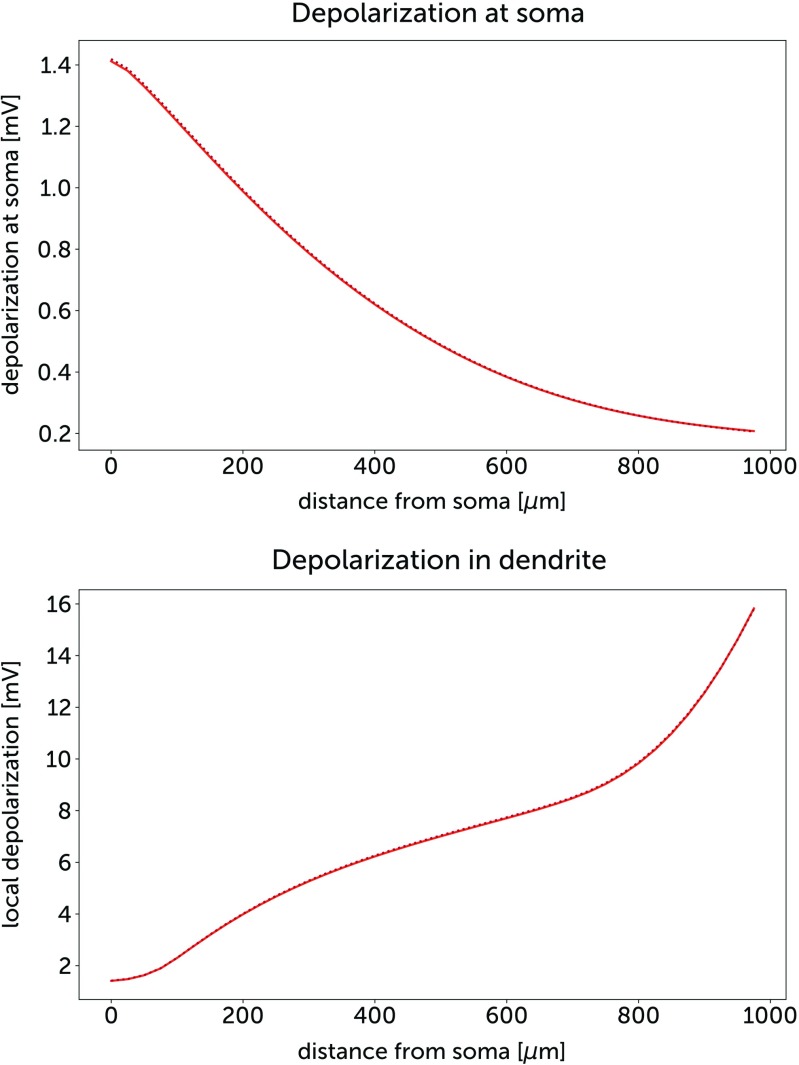
Depolarization in soma and dendrites following synaptic input. *Top:* The depolarization in soma for synaptic inputs at different dendritic locations. *Bottom:* The local dendritic depolarization is shown for the same synaptic stimuli. The synaptic conductance was 0.5 nS

We used a model in which a somatic compartment was connected with a linear dendrite. The morphological and electrophysiological parameters were chosen to obtain the realistic membrane depolarization at soma and local membrane depolarization as a function of distance of a synapse from the soma (Spruston 2008), [see Appendix, Fig. 8]. The diameter of a soma was 40 *μm* and the diameter of the dendrite was gradually decreasing with the distance from soma. In most of simulations there were 200 dendritic compartments of the length 5 *μ*m; for Fig. 5c there were 50 dendritic compartments of length 20 *μ*m.

Voltage-gated fast sodium and delayed-rectifier potassium ion channels were present in the soma and dendrite. The cable equation was as follows:

1 $$\begin{array}{@{}rcl@{}} c_{m} \frac{\partial V}{\partial t} & = & \frac{d}{4 r_{i}} \frac{\partial^{2} V}{\partial x^{2}} + g_{L}(E_{L} - V) \\ && + g_{Na} m^{3} h (E_{Na} - V) + g_{K} n^{4} (E_{K} - V) \\ && + g_{e}(x,t)(E_{e} - V) + g_{i}(x,t)(E_{i} - V) \end{array} $$

where *c*_*m*_ is a specific capacitance, *d* is a diameter of a dendrite, *r*_*i*_ is an intracellular specific resistance. *g*_*L*_ is a leak conductance, *E*_*L*_ is a leak reversal potential, *g*_*Na*_ is a maximal conductance of fast sodium channels, *E*_*Na*_ is a reversal potential of sodium current, *g*_*K*_ is a maximal conductance of potassium current, *E*_*K*_ is a reversal potential of a potassium current. *g*_*e*_, *E*_*e*_ and *g*_*i*_, *E*_*i*_ are conductances and reversal potentials of excitatory and inhibitory synapses respectively. *m*, *h* and *n* represent gate states of ion channels. The gate states evolves according to equations:

2 $$\begin{array}{@{}rcl@{}} \frac{dm}{dt} &=& \alpha_{m} (1 - m) - \beta_{m} m \\ \frac{dh}{dt} &=& \alpha_{h} (1 - h) - \beta_{h} h \\ \frac{dn}{dt} &=& \alpha_{n} (1 - n) - \beta_{n} n \end{array} $$

where

$$\begin{array}{@{}rcl@{}} \alpha_{m} &=& \frac{ - 0.32 \frac{1}{\text{mV}} (V - V_{th} - 13 \text{mV})}{\exp(-\frac{V - V_{th} - 13 \text{mV}}{4 \text{mV}}) - 1} \\ \beta_{m} &=& \frac{ 0.28 \frac{1}{\text{mV}} (V - V_{th} - 40 \text{mV})}{\exp(\frac{V - V_{th} - 40 \text{mV}}{5 mV}) - 1} \\ \alpha_{h} &=& 0.128 \exp \left( - \frac{V - V_{th} - 17 \text{mV}}{18 \text{mV}} \right) \end{array} $$

3 $$\begin{array}{@{}rcl@{}} \beta_{h} &=& \frac{4}{1 + \exp(-\frac{V - V_{th} - 40 \text{mV}}{5 \text{mV}})} \\ \alpha_{n} &=& \frac{ - 0.032 \frac{1}{\text{mV}} (V - V_{th} - 15 \text{mV})}{\exp(-\frac{V - V_{th} - 15 \text{mV}}{5 \text{mV}}) - 1} \\ \beta_{n} &=& 0.5 \exp \left( - \frac{V - V_{th} - 10 \text{mV}}{40 \text{mV}} \right) \end{array} $$

Whenever there is an excitatory (e) or inhibitory (i) synaptic event: *g*_*s*_(*t*) → *g*_*s*_(*t*) + *δg*_*s*_(*t*). The value of *δg*_*s*_(*t*) is the weight of a synapse. Between events, synaptic conductances follow the equation:

4 $$\begin{array}{@{}rcl@{}} \frac{dg_{s}}{dt} = - \frac{1}{\tau_{s}} g_{s}(t) \end{array} $$

where *τ*_*s*_ is a time constant of synaptic current.

**Fig. 9:**
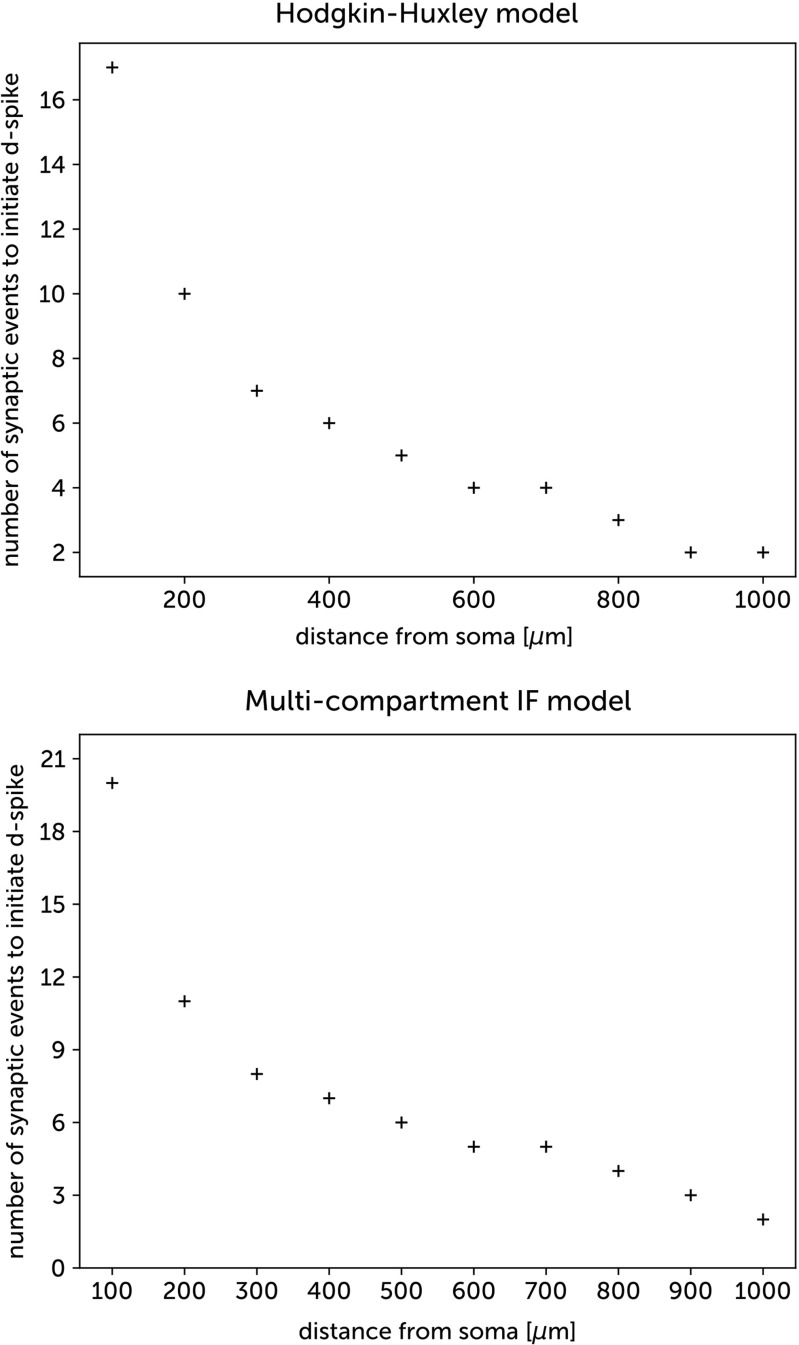
Number of synaptic events needed to initiate a dendritic spike in a Hodgkin-Huxley model and in a multi-compartment integrate and fire model. The synaptic conductance was equal to 0.5 nS for both models

The number of simultaneous synaptic events needed to initiate a dendritic sodium spike depends on the distance between a synapse and the soma, [see Appendix, Fig. 9]. Few synaptic events are needed to initiate sodium spike in the distal tip of the dendrite. This aspect of dendritic sodium spike initiation was studied in more detailed computational models (Destexhe et al. 2003).

**Fig. 10:**
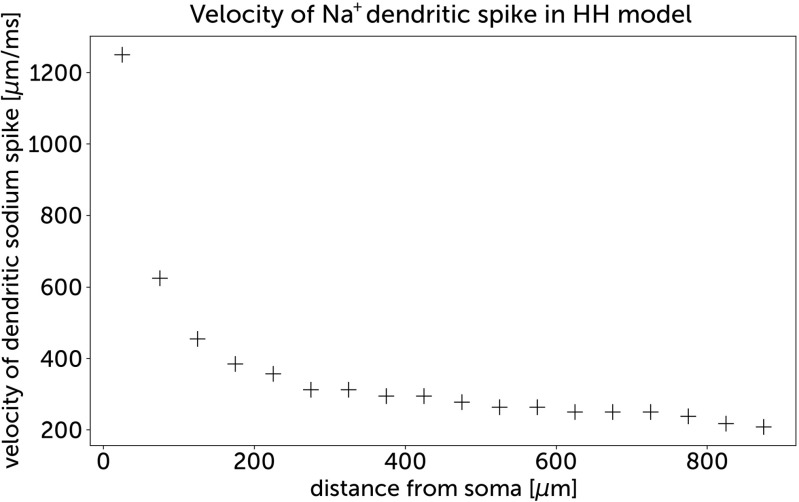
Velocity of dendritic sodium spikes as a function of the distance from the soma in a Hodgkin-Huxley model. Dendritic sodium spikes accelerate while propagating toward the soma

The velocity of dendritic sodium spikes decreases with the distance from the soma starting with 1200 *μ*m/ms in the proximal dendrite to 200 *μ*m/ms in the distal dendritic end (Fig. 10), similar to experimental observations (Turner et al. 1994).

In our simulations: *c*_*m*_ = 1 *μ* F/cm^2^, *r*_*i*_ = 100 Ωcm, *d* = 1 *μ* m, *g*_*L*_ = 100 *μ* S/cm^2^, *E*_*L*_ = − 70 mV, *g*_*Na*_ = 12 mS/cm^2^, *E*_*Na*_ = 58 mV, *g*_*K*_ = 7 mS/cm^2^, *E*_*K*_ = − 80 mV, *V*_*th*_ = − 63 mV, *E*_*e*_ = 0 mV, *E*_*i*_ = − 75 mV, *τ*_*s*_ = 5 ms.

### Integrate and fire dendritic model

We used an exponential integrate and fire model, which can be easily extended by neural adaptation mechanism, forming an AdEx model (Brette and Gerstner 2005). In the exponential integrate and fire model a spike is triggered by the exponential depolarizing current (Fourcaud-Trocmé et al. 2003), which provides a smooth spike initiation zone in place of the fixed threshold of the Leaky Integrate and Fire (LIF) model. For a multi-compartment model a membrane voltage *V* (*t*, *x*) is governed by a following cable equation:

5 $$\begin{array}{@{}rcl@{}} c_{m} \frac{\partial V}{\partial t} \!&=&\! \frac{d}{4 r_{i}} \frac{\partial^{2} V}{\partial x^{2}} + g_{L}(E_{L} - V) + g_{L} {\Delta}_{T} \exp \left( \frac{V - V_{T}}{{\Delta}_{T}}\right) \\ &&+ g_{e}(x,t)(E_{e} - V) + g_{i}(x,t)(E_{i} - V) \end{array} $$

where *V*_*T*_ is a spike threshold and Δ_*T*_ is a slope factor.

This equation is solved numerically by discretization of the equations over multiple dendritic compartments. When a membrane potential of any compartment is near threshold *V*_*T*_ depolarizing exponential current surpasses other currents and membrane voltage quickly tends to infinity. Whenever the membrane potential crosses a peak value *V*_*p*_ we detect a spike and the compartment enters into a refractory period in which voltage is repolarized according to the equation:

6 $$ c_{m} \frac{dV}{dt} = - \frac{1}{\tau_{r}}(V-E_{L}) $$

The exponential decay characteristic of this equation models the falling phase of an action potential. The time constant of repolarization *τ*_*r*_ was chosen in such a way that after 1 ms the difference between *V* and *E*_*L*_ was smaller than 0.01 mV. After emitting a spike the compartment stays in the refractory state for time *t*_*r*_.

The number of simultaneous synaptic events needed to initiate a dendritic spike depends on the distance between the synapse and soma, [see Appendix, Fig. 9]

In our simulations: *c*_*m*_ = 1 *μ* F/cm^2^, *r*_*i*_ = 100 Ωcm, *d* = 1 *μ* m, *g*_*L*_ = 100 *μ* S/cm^2^, *E*_*L*_ = − 70 mV, *V*_*T*_ = − 50 mV, *V*_*p*_ = − 20 mV, *τ*_*r*_ = 117 *μs*/*c*_*m*_, Δ_*T*_ = 2 mV, *E*_*e*_ = 0 mV, *E*_*i*_ = − 75 mV, *τ*_*s*_ = 5 ms.

### Simulation of the discrete-state model

Given a set $\mathcal S$ of somatic inputs, we describe our procedure to re-construct the propagating dendritic fronts leading to somatic spikes. Take the latest element *s*_1_ = (*t*_1_, *x*_1_) ∈ *S*. We look for possible annihilation events with its outgoing front, i.e. going away from the soma located at *x* = 0. Hence, we select all inputs *S*^*↑*^(*s*_1_) in *S* ∖{*s*_1_} which are located in the cone above *s*_1_ defined as *S*^*↑*^(*s*_1_) ≡{(*t*, *x*) : *x* ≥±(*t* − *t*_1_) + *x*_1_}. Consider the somatic input *s*_2_ ∈ *S*^*↑*^(*s*_1_) closest to the line *x* = −(*t* − *t*_1_) + *x*_1_: it will produce an annihilation event with *s*_1_. Perform the same for *s*_2_ recursively. This gives a chain of annihilation events (*s*_1_, *s*_2_,..., *s*_*k*_). We do the same for possible annihilation events with the ongoing front from *s*_1_, i.e. going toward the soma located at *x* = 0. It gives another chain of events (*s*_−*p*_,..., *s*_0_, *s*_1_). Hence, we found a chain of annihilation events *C*(*s*_1_) = (*s*_−*p*_,..., *s*_*k*_): it produces **exactly one** somatic spike. We continue the same procedure with *S* ∖ *C*(*s*_1_) until *S* = *∅*. The overall procedure runs in *O*(*n*^2^) where *n* is the cardinal of *S*.

### Correlation measure for spike trains

To measure the correlation between two spike trains *S*_*i*_(*t*) and *S*_*j*_(*t*) we applied the cross-covariance function CCVF_*ij*_(*s*) = 〈*S*_*i*_(*t*)*S*_*j*_(*t* + *s*)〉−〈*S*_*i*_(*t*)〉〈*S*_*j*_(*t*)〉, where 〈...〉 denotes a time average. The normalized integral $ C_{{\Delta } t}^{ij} = \frac {1}{\langle S_{i}(t) \rangle } {\int }_{-{\Delta } t}^{{\Delta } t} \text {CCVF}_{ij}(s) ds$ tells us about a correlation within a time window [−Δ*t*,Δ*t*] [Fig. 1].

## Appendix:

In this appendix, we show additional properties related to the dendritic models. The profile of depolarization, in soma and dendrite, caused by a synaptic input is shown in Fig. 8. The numbers of synaptic inputs needed to initiate a dendritic spike are shown in Fig. 9, and the velocity of sodium dendritic spikes in Fig. 10.
